# Diversified clinical presentations associated with a novel *sal-like 4* gene mutation in a Chinese pedigree with Duane retraction syndrome

**Published:** 2013-05-06

**Authors:** Ming-ming Yang, Mary Ho, Henry H.W. Lau, Pancy O.S. Tam, Alvin L. Young, Chi Pui Pang, Wilson W.K. Yip, LiJia Chen

**Affiliations:** 1 Department of Ophthalmology and Visual Sciences, the Chinese University of Hong Kong, Hong Kong, China; 2 Department of Ophthalmology, Prince of Wales Hospital, Hong Kong, China

## Abstract

**Purpose:**

To determine the underlying genetic cause of Duane retraction syndrome (DRS) in a non-consanguineous Chinese Han family.

**Methods:**

Detailed ophthalmic and physical examinations were performed on all members from a pedigree with DRS. All exons and their adjacent splicing junctions of the *sal-like 4* (*SALL4*) gene were amplified with polymerase chain reaction and analyzed with direct sequencing in all the recruited family members and 200 unrelated control subjects.

**Results:**

Clinical examination revealed a broad spectrum of phenotypes in the DRS family. Mutation analysis of *SALL4* identified a novel heterozygous duplication mutation, c.1919dupT, which was completely cosegregated with the disease in the family and absent in controls. This mutation was predicted to cause a frameshift, introducing a premature stop codon, when translated, resulting in a truncated SALL4 protein, i.e., p.Met640IlefsX25. Bioinformatics analysis showed that the affected region of SALL4 shared a highly conserved sequence across different species. Diversified clinical manifestations were observed in the c.1919dupT carriers of the family.

**Conclusions:**

We identified a novel truncating mutation in the *SALL4* gene that leads to diversified clinical features of DRS in a Chinese family. This mutation is predicted to result in a truncated SALL4 protein affecting two functional domains and cause disease development due to haploinsufficiency through nonsense-mediated mRNA decay.

## Introduction

Duane retraction syndrome (DRS) is a congenital disorder of ocular motility, which is pan-ethnic and accounts for approximately 1% of the total cases of strabismus [1]. Individuals with DRS typically manifest horizontal eye movement limitation, globe retraction with palpebral fissure narrowing in attempted adduction. DRS can be classified into types I, II, and III, with the presence of abduction, adduction, or both, respectively [2]. Occurrence of DRS is usually sporadic. Reported familial DRS is mostly bilateral with vertical movement abnormalities and autosomal dominant inheritance [3]. In addition, several associated systemic manifestations have been described in patients with DRS, most commonly involving facial anomalies, hearing dysfunction, vertebral column anomalies, and variable degrees of limb malformations [4-7]. The exact etiology of DRS is still elusive. Electromyographic and magnetic resonance imaging (MRI) studies suggested that DRS might result from abnormal development of the abducent nerve (sixth cranial nerve) [8,9].

Linkage analyses of DRS have successfully mapped its associated loci to chromosomes 2q13, 4q27, 8q13, 22q11, and 20q13 [10-13]. Recently mutations of the *sal-like 4* (*SALL4*; MIM# 607343) gene on chromosome 20 have been linked to DRS or DRS associated with radial forearm malformations, also known as Okihiro syndrome [14-17]. *SALL4*-related disorders include Duane-radial ray syndrome (DRRS, Okihiro syndrome) and acro-renal-ocular syndrome (AROS). Okihiro syndrome, characterized by radial malformation associated with Duane congenital abnormalities, is used interchangeably with DRRS. Another *SALL-4* related syndrome is known as acro-renal-ocular syndrome (AROS), characterized by radial ray malformation, Duane abnormality, renal malformation (mild malrotation, ectopia, horseshoe kidney, renal hypoplasia, vesicoureteral reflux, bladder diverticula), and coloboma [14]. More than 20 mutations in *SALL4* have been reported in association with these disorders. The *SALL4* gene is located in chromosome 20q13–20q13.2, with a length of 18.14 kb and 4 exons, encoding for a 1053 amino-acid-residue protein. *SALL4*, a new member of the SAL family of proposed C2H2 zinc finger transcription factors, plays important transcription roles during human embryogenesis [15,18]. *SALL4* is actually the first identified disease-causing gene of DRS and likely plays a crucial role in the development of the abducens motoneuron. Although several mutations have been reported in *SALL4*, disease phenotypes vary greatly among different *SALL4* mutations, and the phenotype–genotype correlation remains elusive. In this paper, we report for the first time a Chinese family with members presenting with isolated DRS or DRS associated syndromes in a dominant trait.

## Methods

This study was approved by the Research Ethics Committees of the Chinese University of Hong Kong and conducted in accordance with the tenets of the Declaration of Helsinki. Informed consent was obtained from each participant after detailed explanation of the nature of the study.

### Study subjects

**Figure 1:**
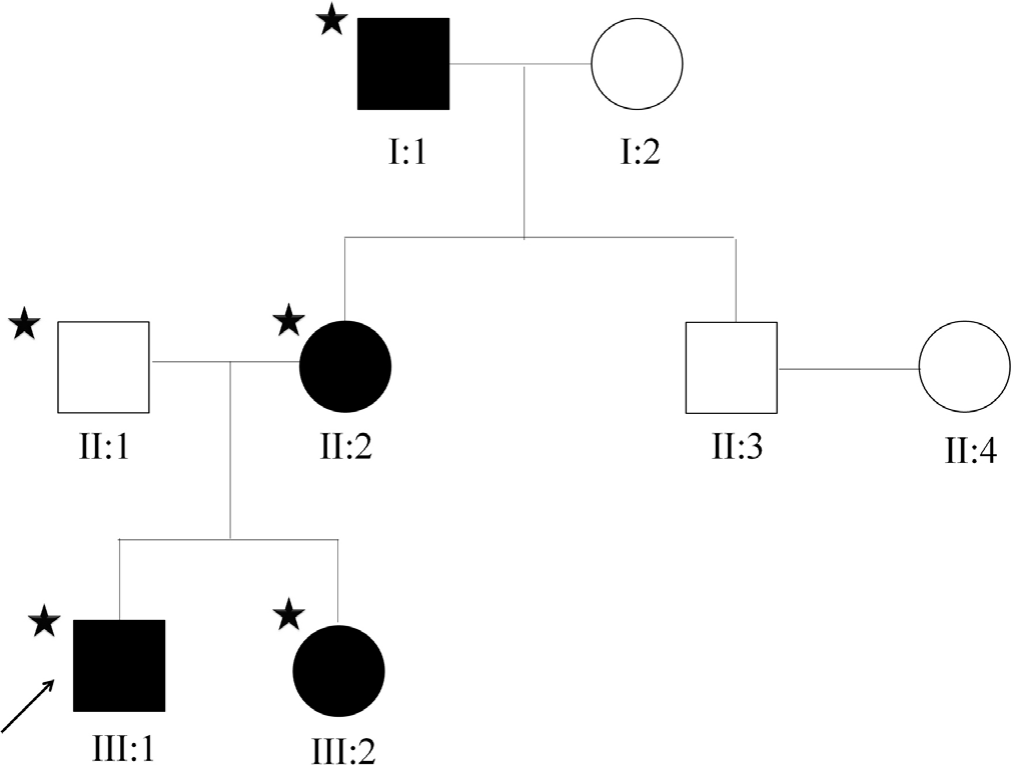
Pedigree of a Chinese family with Duane retraction syndrome. Squares: men; circles: women; filled symbols: affected; empty symbols: normal individual; arrow: the proband; star: DNA samples available for this study.

A three-generation Chinese Han family with DRS was recruited at the Department of Ophthalmology, Prince of Wales Hospital, Hong Kong (Figure 1). Affected status was determined with detailed ophthalmic examinations that included visual acuity, refraction, intraocular pressure, slit-lamp evaluation, and ocular motility testing. Additionally, systemic tests including audiometry and neurologic testing were performed. Detailed medical and family histories were also documented. Peripheral blood was collected from five available members of the family, including I:1, II:1, II:2, III:1, and III:2 (Figure 1). Peripheral blood was collected using EDTA tubes from five available members of the family, and stored in -80 °C before processing for genomic DNA isolation.

Also included in this study was a group of 200 unrelated Chinese control subjects (age >40 years) who had undergone complete ocular examination and were confirmed to be free of major ocular and systemic disorders. Peripheral blood samples were collected from each control subject after informed consent was received.

### Mutation screening

Genomic DNA was extracted from whole blood using the QIAmp Blood kit (Qiagen, Hilden, Germany) according to the supplier's instructions. All exons and intron-exon boundaries of *SALL4* were amplified with PCR and analyzed with direct sequencing in all study subjects. In total, seven pairs of primers, designed by using Primer3 (accessed June 1, 2012) referring to the sequence of human *SALL4* gene from the Ensembl database (accessed June 1, 2012), were used (Table 1). Targeted regions were amplified by PCR using 20 ng of genomic DNA in a 25 μl reaction mixture containing 0.2 μM primers, 0.2 mM deoxyribonucleotide triphosphate, 1 U Taq DNA polymerase (Invitrogen, Carlsbad, CA), and 1.5/2.0 mM MgCl_2_ (indicated in Table 1). The PCR thermal cycling conditions were initial denaturation at 95 °C for 2 min followed by 45 cycles of 95 °C for 40 s, 58/60 °C for 40 s (annealing Tm for each primer is indicated in Table 1), and 72 °C for 40 s, with a final extension step of 72 °C for 10 min. Bidirectional sequencing of PCR products was performed using the BigDye Terminator Cycle Sequencing (v3.1) protocol in an ABI 3130×l Genetic Analyzer automated sequencer (Applied Biosystems, Foster City, CA). All mutation descriptions followed the nomenclature recommendations of the Human Genomic Variation Society (HGVS).

**Table 1:** Primer Sequence and Thermal Cycling Condition for Genotyping *SALL4*

**Amplicon**	**Forward Primer (5′ – 3′)**	**Reverse Primer (5′ – 3′)**	**Size (bp)**	**Ta (**°C**)**	**MgCl_2_ (mM)**
1	TCAGGGCTCATGATAAATCG	AATCTCGGCTCCTGAATTTG	402	58	2.0
2A	GATTATAGATGTGAGCGACGGTGC	CTTCCAGCTTTCTGGCTGAG	795	60	1.5
2B	TACAGCAGATCCAGCTCACC	GCCACTTTGTCCTGGAACTC	673	60	2.0
2C	TGGGACTGATAGCTCCTTGC	ACCCCAAGGTGTGTCTTCAG	672	60	1.5
2D	TCAGAGCTCCCTCAAGATGC	CAGGCTCCTTTTTGATGACC	791	58	2.0
3	ACAAAGCCAGCTCCAGACTC	CGGCTTGTGCCAATAAGAAG	471	60	2.0
4	ATTCTTGGCTTGCCAGTGAG	TGTGTCTGCATTGCTCCTTC	522	60	2.0

Ta, Annealing temperature.

### Bioinformatics analysis

Sequences of SALL4 orthologs in other vertebrate species were retrieved from the NCBI Reference Sequence database (accessed June 23, 2012). Multiple alignments of SALL4 orthologs from different vertebrate species were conducted using a Web-based program, T-Coffee (ver. 7.71, provided in the public domain by the Center for Genomic Regulation, Barcelona, Spain, accessed June 23, 2012).

## Results

### Clinical characteristics of the study subjects

As illustrated in Figure 1, this family is represented by three generations of individuals. Five members consented to participate in this study, of whom four are affected with isolated DRS or combined with bone abnormalities.

**Figure 2:**
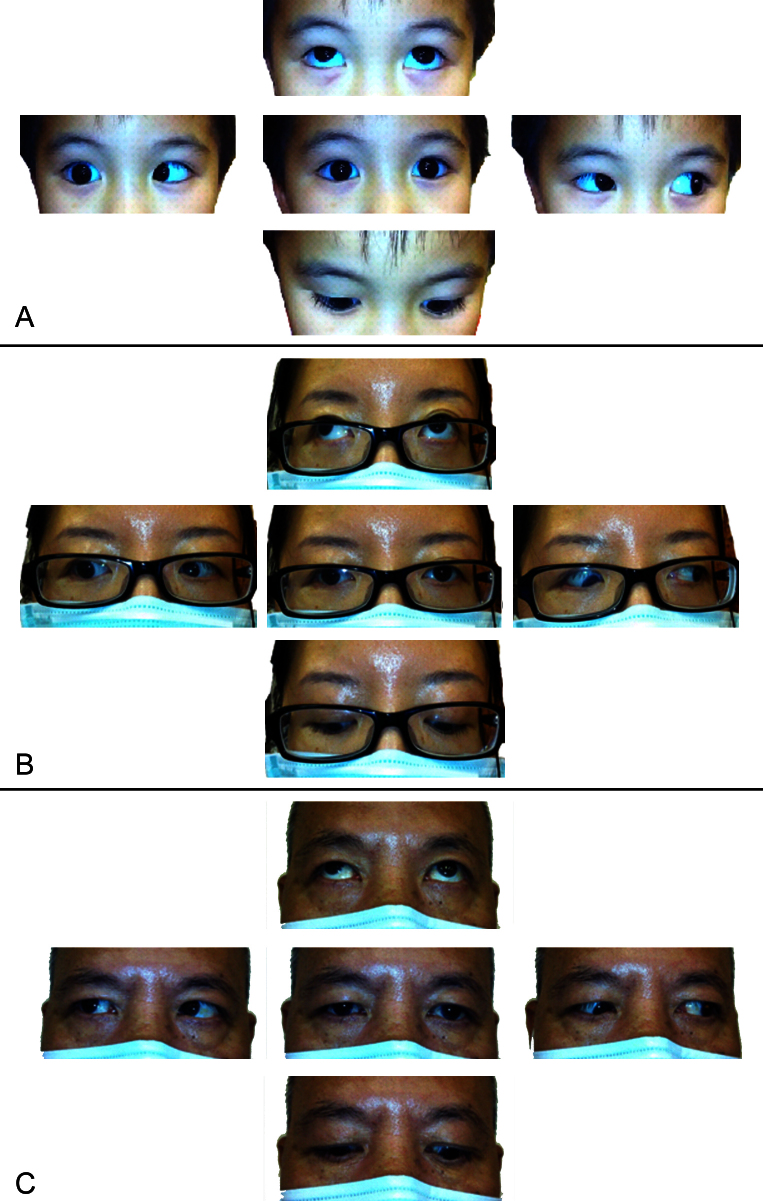
Clinical pictures showing photos of ocular gazes in proband III:1 (**A**: Four-year-old boy), II-2 (**B**. his mother) and I-1 (**C**. his grandfather). All shared similar features with limitation in right sided abduction, upshooting or downshooting of right eye with narrowing of palpebral fissure on attempted adduction gaze.

**Figure 3:**
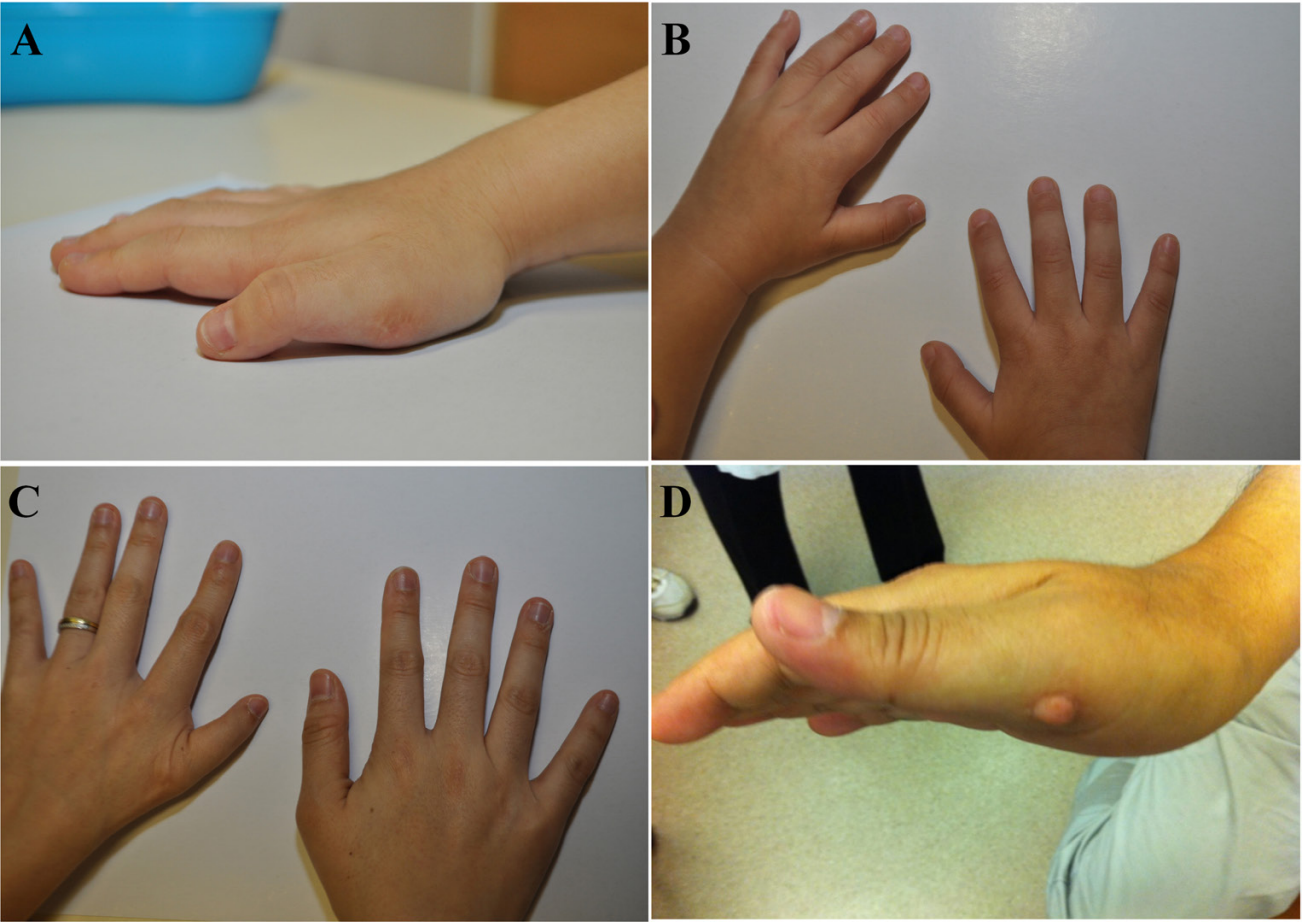
Phenotypic features of patients. **A**: A 4-year-old boy (III:1) with left hand scar at thenar prominence at the site of previous surgery to excise of duplication of thumb. **B**: A 6-month-old girl (III:2) has no signs of limb malformations. **C**: A 36-year-old woman (II:2) with residual signs of a previous extra left thumb , and left thenar hypoplasia and hypoplasia of thumb. **D**: A 60-year-old man (I:1) with residual incomplete excision of an extra right thumb.

The proband (III:1) was a 4-year-old boy, who was referred to us by his pediatrician for suspected convergent squint. He was born with an uncomplicated normal delivery and enjoyed normal development and visual acuity. Ophthalmic examination showed that he had right eye abduction weakness and noticeable narrowing of palpebral fissure on attempted abduction gaze. He also presented with right esotropia at the primary position of gaze (Figure 2A). Other ocular examinations including refraction were unremarkable. On physical examination, a surgical scar was noted at the medial side of his right thumb, which was created after excision of his duplicated thumb (preaxial polydactyly; Figure 3A). Hearing test, cardiac examinations, limb examinations, and ultrasound scans of the kidneys were normal.

**Figure 4:**
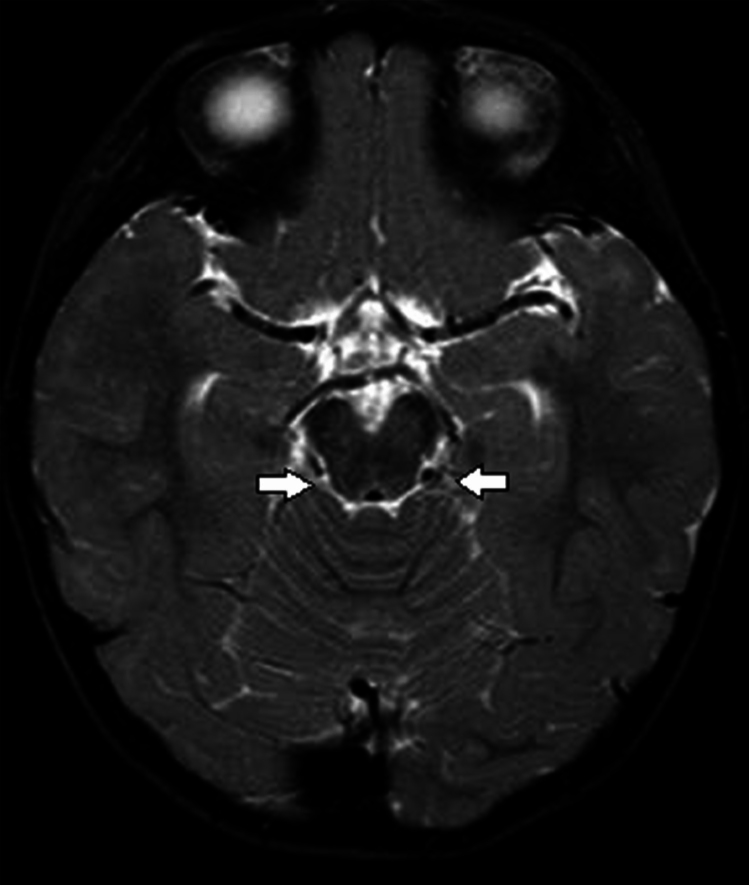
Magnetic resonance imaging of the brain of proband III:2. The thin linear structure coursing obliquely from the left pontomedullary region in the left pontine cistern suggests the left abducens nerve, which was absent in the right side.

The proband’s younger sister (III:2), a 6-month-old girl, was referred at the same time to our clinic for convergent squint. She was born full term, with normal prenatal and postnatal checkups. Ophthalmic examination showed that she had large angle convergent squint at the primary position of gaze. Narrowing of bilateral palpebral fissure was noticed on both sides in attempted abduction. MRI was performed to rule out any lesion occupying intracranial space causing bilateral sixth nerve palsy. MRI of the brain was normal, except the right abducens nerve was absent (Figure 4). The brain MRI did not have information on the nuclei. Cycloplegic refraction showed mild hypermetropia. Physical examination showed no signs of upper limb malformations (Figure 3B). In view of the esotropia in the primary position, bilateral medial rectus recession was performed. Her preoperative renal function tests were normal. Their mother (II:2), a 36-year-old woman, was found to have similar problems. She had right-sided DRS (Figure 2B) and left preaxial duplication of the thumb. Additional features including hypoplasia of the thenar muscle and hypoplasia of the left thumb were also noted (Figure 3C). Their grandfather (I:1), a 72-year-old man, had a similar clinical presentation as his daughter (II:2; Figure 2C and Figure 3D). Examination and further history-taking revealed no history of cardiac abnormality, hearing problems, or skeletal malformations. Medical records including renal function tests were retrieved for the mother (II:2) and grandfather (I-1), which were normal. Other members of the family did not have any clinical signs or symptoms suggestive of DRS. In summary, all four affected family members have unilateral or bilateral Duane features with or without evidence of radial-ray abnormality, and clinical evidence of other abnormalities including hearing, cardiac, or renal malformation was absent, supporting clinical diagnosis of DRRS (Okihiro syndrome).

### Mutation analysis

**Figure 5:**
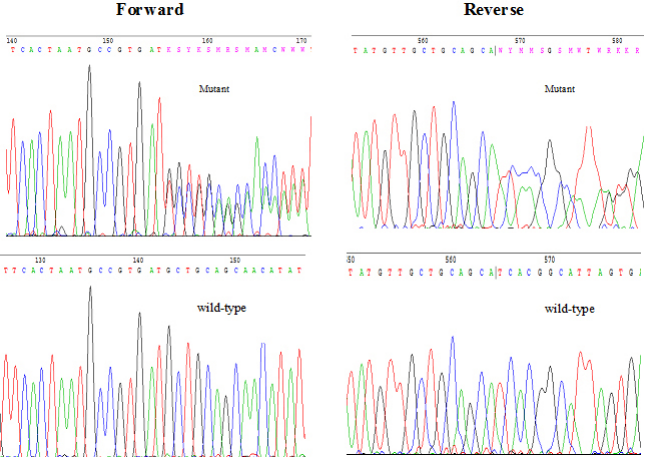
Sequence chromatographs of the novel heterozygous mutation c.1919dupT in *SALL4* and the respective wild-type sequence.

Direct sequencing was performed to cover all exons and intron-exon boundaries of the *SALL4* gene. A heterozygous duplication c.1919dupT (Figure 5) in exon 2 was identified exclusively in all affected family members but not in any of the unaffected family members. The duplication was absent in the 200 unrelated controls. No other variants were detected in the controls. The c.1919dupT mutation was predicted to cause a frameshift introducing a premature stop codon after 73 bp in the messenger RNA, which encodes a truncated SALL4 protein (p.M640IfsX25), affecting two zinc finger domains.

**Figure 6:**
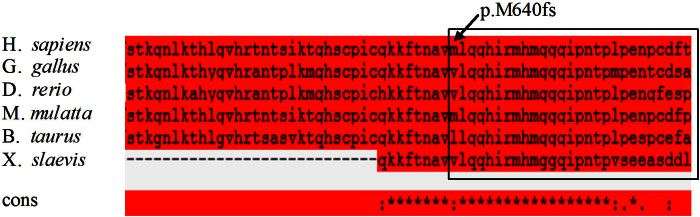
Multiple alignment of the SALL4 protein sequence (partial) from different species. The box indicates that the deleted region affected by the mutation (p.Met640IlefsX25) in SALL4 was highly conserved.

To evaluate the functional significance of this mutation, multiple alignments of SALL4 sequences were applied to compare the sequences of human, chicken, zebrafish, rhesus monkey, cattle, African clawed frog. The results showed that the affected region of SALL4 was highly conserved across species and revealed the strong identity of the deleted region of protein at SALL4 zinc finger domains (Figure 6).

## Discussion

In this study, we identified a novel *SALL4* mutation in a Chinese family with DRS presenting with variable phenotypes. The mutation showed complete disease cosegregation and is highly likely to be disease-causing, although further functional characterization is needed to confirm the mutation’s role in disease etiology. Autosomal dominant DRS has a high penetrance, although considerable variability within a pedigree also exists. This may explain the wide-spectrum phenotypes observed in the family evaluated. Until now, several sporadic cases or pedigrees and mutations implicated in DRS have been reported in Caucasians. To the best of our knowledge, this is the first genetic study, and the first pedigree report, on DRS in the Chinese population.

**Figure 7:**
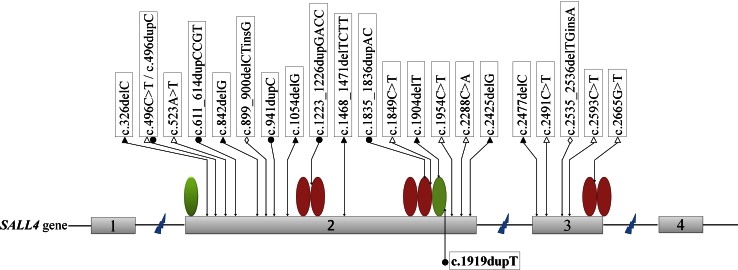
Schematic representation of the SALL4 gene/protein and localization of all mutations identified in Duane-related syndromes. Oval symbols: zinc finger domain. Symbols of mutations: open triangle, nonsense; black triangle, deletion; black circle; duplication; open diamond, combined deletion-insertion. Previously found mutations are above the schematic gene, and the mutation (c.1919dupT) reported in this study is under the schematic gene.

Mutations in the *SALL4* gene have been shown to cause DRS and DRS-associated disorders, namely, Okihiro syndrome, acro-renal-ocular syndrome, Holt-Oram syndrome, or suspected thalidomide embryopathy [19-22]. Several overlapping phenotypes are shared among these disorders. Okihiro syndrome, an autosomal dominant disorder characterized by radial ray defects combined with Duane anomaly, is the most common condition known to overlap clinically with DRS. The *SALL4* gene consists of four coding exons and encodes a protein with three highly conserved C2H2 double zinc finger domains, the second of which has a single C2H2 zinc finger attached at its C-terminal end, as well as an N-terminal C2HC zinc finger motif [15,18,23,24]. To date, 22 *SALL4* mutations have been described in Duane-related syndromes, especially Okihiro syndrome [14-17,21,23]. Seventeen are located in exon 2, and five within exon 3. These known mutations are nonsense mutations, duplications, or deletions (Figure 7).

The c.1919dupT mutation identified in this study was a duplication located in exon 2. This mutation is predicted to generate a frameshift resulting in a truncated protein denoted as p.Met640IlefsX25. Thus, when translated, the premature termination codon would truncate the SALL4 polypeptide by 414 residues, i.e., appropriately 40% of the total length. The codon would truncate the second C2H2 double zinc finger domain and lead to a complete deletion of the third domain due to premature termination. The region containing these three functional domains was conserved; our sequence analysis also showed the deleted residues in the second domain of SALL4 to be highly conserved across different vertebrate species. This heterozygous c.1919dupT mutation plays a role in the disease pathogenesis via haploinsufficiency through a pathway known as nonsense-mediated mRNA decay, an mRNA surveillance mechanism found in all eukaryotic organisms that leads to a degradation of the transcripts with introns in the 3′ untranslated region. The mutation subsequently prevents the synthesis of truncated proteins that may have toxic effects such as dominant negative interactions [25,26]. In fact, all the mutations identified thus far in *SALL4* associated with Duane-related syndromes are likely to be disease-causing via haploinsufficiency [27]. This proposition was based on the observation that disease-causing deletions of all coding exons in two independent families that explicitly resulted in haploinsufficiency [16]. The c.1919dupT mutation described in our Chinese family with DRS provides further evidence supporting that *SALL4* mutations affect the disease phenotype through nonsense-mediated mRNA decay.

To date, there is insufficient information to correlate *SALL4* mutations with the severity of the phenotypes of DRS and DRS-associated syndromes. Although the mutation p.R865X has been reported to cause either a mild or severe phenotype [21], c.1919dupT in this present family gives clear variations in clinical features. Varying degrees of ocular disorders and limb involvements were manifested in the c.1919dupT carriers. Such complex disease manifestation might have been modified by local epigenetics or other mutations that have yet to be identified.

Kohlhase et al. demonstrated that the expression of SALL4 in human could be detected only in the testis and ovary [18]. In animal studies, *SALL4* mRNA expression has been detected in the embryo and well described during different developmental periods; the expression in the progress zone of the limb buds fitted well with the radial ray anomalies in patients with Duane-related syndromes [18]. In addition, a reduced dosage of SALL4 in the mid-hindbrain region was expected to result in disturbed development of the sixth cranial nerve [18]. Two independent studies noted no mutations in *SALL4* could be detected in isolated DRS cases, suggesting other genetic influences in addition to *SALL4* [28,29]. Recently, mutations in *CHN1* have been found in association with isolated families with DRS [30].

In summary, we have identified a novel heterozygous *SALL4* mutation cosegregating with DRS in a Chinese pedigree. This mutation is predicted to result in a truncated SALL4 protein affecting two functional domains, and haploinsufficiency due to nonsense-mediated mRNA decay may be responsible for the disease pathogenesis. The results of this study provide further evidence for understanding the genetic causes of DRS and extend its phenotypic and mutational spectra.
